# The Potential Role of Microbiota in Age-Related Cognitive Decline: A Narrative Review of the Underlying Molecular Mechanisms

**DOI:** 10.3390/ijms26041590

**Published:** 2025-02-13

**Authors:** Piotr Olejnik, Aleksandra Golenia, Jolanta Małyszko

**Affiliations:** 1 Department of Neurology, Medical University of Warsaw, 02-097 Warsaw, Poland; piotrek.olejnik2001@gmail.com (P.O.); aleksandra.golenia@wum.edu.pl (A.G.); 2 Department of Nephrology, Dialysis and Internal Medicine, Medical University of Warsaw, 02-097 Warsaw, Poland

**Keywords:** aging, age-related cognitive impairment, microbiota, gut-brain axis, neurodegeneration

## Abstract

As the world’s population continues to age, social patterns are changing, making aging a notable public health challenge. With aging as the major risk factor for cognitive decline, the global prevalence of dementia is projected to triple in the next 25 years. In light of the growing body of evidence of the involvement of microbiota in health and pathology, its role in age-related cognitive decline should be explored. Therefore, the aim of this narrative review is to thoroughly analyze the ways in which microbiota might affect the aging process and age-related cognitive decline. Overall, aging is a complex phenomenon manifested at systemic, cellular and molecular levels. According to recent studies, gut microbiota composition may influence age-related changes through the gut-brain axis. One mechanism involves dysbiosis-related chronic systemic inflammation, leading to the blood-brain barrier disruption and subsequent neuroinflammatory processes. In addition to inflammaging, gut microbiota may induce oxidative stress, which is another key factor in brain aging. Finally, not only gut microbiota, but also microbiota colonizing the oral cavity may be associated with age-related neurodegenerative diseases.

## 1. Introduction

Until coronavirus disease 2019 (COVID-19) pandemic, the life expectancy had been on a steady rise from 45.7 years in 1950 to 72.6 years in 2019 [1]. According to the United Nations Population Division, the number of centenarians worldwide increased from 96,000 in 1990 to as many as 451,000 in 2015 [2]. The world’s global population is continuously aging, resulting in changes in social structures of not only developed, but also developing countries [3,4]. Population aging can be defined as an increase in the percentage of people over the age of 60, and a corresponding decrease in the percentage of people under the age of 15 [3]. For instance, in 1950, no country had more than 11% of its population aged 65 and over, while in a hundred years, by 2050, this figure might reach 38% [4]. Since age is widely considered a primary risk factor for cognitive impairment (CI) [5], it is not surprising that the global prevalence of dementia could triple by that time [6]. López-Otín et al. distinguish 12 indicators of mammalian aging, including genetic factors, loss of proteostasis, impaired macroautophagy, mitochondrial dysfunction, altered intercellular communication, chronic inflammation, or dysbiosis. The authors also classify them into three subgroups: primary, antagonistic, and integrative [7]. Similarly, Mattson and Arumugam identify more specific features of brain aging involving excessive glial cell activation with neuroinflammation, imbalanced calcium homeostasis, oxidative damage, and impaired molecular waste removal. In addition, the authors highlight the underlying energy imbalance that triggers the pathological changes during aging [8]. Accordingly, it has been found that the aging process and dementia share common features, implying that the two are linked at the molecular level [9]. Moreover, age-related hearing loss and depression increase the risk of CI in the elderly [10,11].

Aging is a complex process involving the accumulation of systemic, cellular and molecular damage throughout an individual’s life. This causes the body to deteriorate and diseases to develop, including cognition impairment [12]. However, because age-related changes are the result of a complex interplay of different processes, it is impossible to explain them by a single isolated pathomechanism [13]. As noted by Leonard Hayflick, two potential modes of age-related changes can be described—one that is associated with specific genetically controlled programs of intentional aging, and the other that is triggered by random and incidental events [14]. Whether the changes are spontaneous or programmed, the lifelong accumulation of DNA mutations significantly increases the risk of developing malignancies [15]. As early as in 1972, Harman developed a theory emphasizing the role of mitochondrial damage in aging. In this theory mitochondria play the role of a biological clock [16]. With a more advanced understanding of the molecular changes in mitochondria, it is now clear that alongside mitochondrial dysfunction, there is an increase in reactive oxygen species (ROS) production, leading to oxidative cellular damage. Moreover, the effects of oxidative stress are exacerbated by a concurrent age-related decline in antioxidant capacity [17]. Research also suggests that aging is associated with inflammation in both the peripheral and the central nervous system (CNS) [18].

Considering that aging is one of the major risk factors for CI [5] and the fact that López-Otín et al. identified dysbiosis—disruption in the balance between the host and its microbiota—amongst key characteristics of brain aging [7], it is accurate to explore how microbiota influences the host organism in health and disease [19]. Also, it seems reasonable to investigate its role in age-related cognitive decline [20]. Additionally, researchers have explored how the gut microbiota may influence both Alzheimer’s disease (AD) [21] and vascular CI [22], the two most common drivers of cognitive dysfunction. These interactions might be particularly important as the composition of microbiota shifts significantly with age, potentially exacerbating or mitigating the development of CI. This narrative review aims to provide a comprehensive overview of how microbiota may influence the process of aging and age-related cognitive decline.

## 2. Methods

A search of the relevant literature was conducted using PubMed and Google Scholar databases to include studies from their inception through 31 December 2024, for a thorough analysis of the topic. The search covered both review papers and research articles, including experimental and clinical studies. Titles and abstracts were screened for terms such as ‘aging’, ‘age-related cognitive decline’, ‘cognitive impairment’, ‘aging molecular basis’, ‘gut microbiota’, ‘oral microbiota’, ‘oxidative stress’, and ‘neurodegeneration’. To ensure comprehensive coverage, a manual review of relevant references was performed. Finally, articles not written in English and items not published as complete scientific papers, for instance conference abstracts, were excluded from our search to ensure the relevance of the review.

## 3. Gut-Brain Axis and Aging

**Figure 1:**
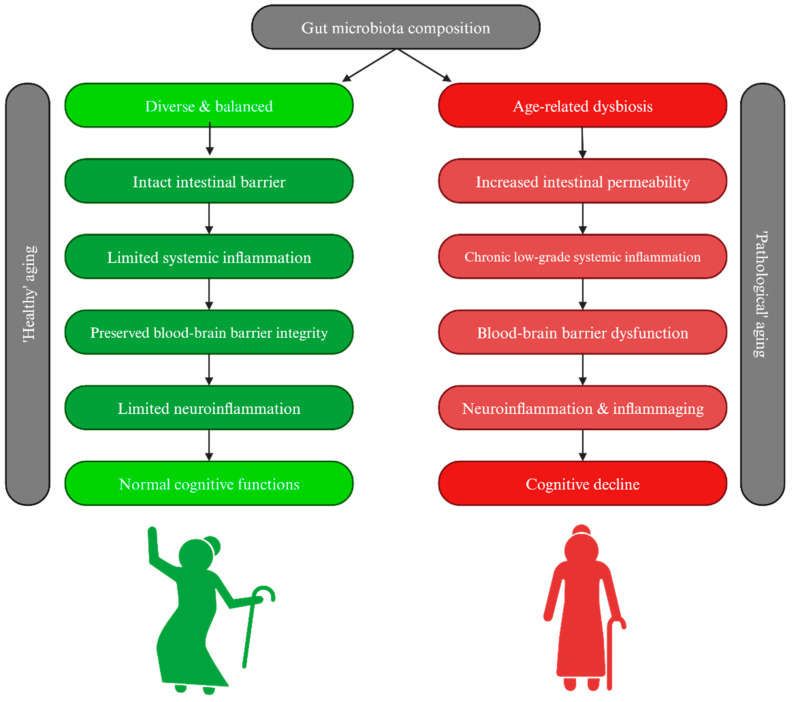
Comparison of healthy and pathological brain aging in relation to gut microbiota composition. In healthy aging (depicted in **left** column), a balanced and diverse gut microbiota supports proper intestinal barrier function, limited systemic inflammatory reactions, maintained blood-brain barrier integrity and consequently preserved normal cognitive function. In comparison, during pathological aging (depicted in **right** column), gut dysbiosis and ‘leaky gut’ might lead to chronic low-grade systemic inflammation, subsequent blood-brain barrier dysfunction, its increased permeability and neuroinflammation, ultimately contributing to cognitive decline.

Microbiota can broadly be described as the collection of trillions of microorganisms, such as bacteria, fungi, protists or archaea, and viruses that colonize the inside and the outside of the human body [23]. Although the terms ‘microbiota’ and ‘microbiome’ are often used interchangeably, their meanings are not entirely synonymous. Microbiome should be defined as the collective genomic composition of microbiota [19]. The composition of microbiota varies depending on the site of colonization [19], and is not constant in the population, but varies in from person to person, as it can be influenced by many factors such as genetic factors, diet, antibiotic use, place of living, and sanitary conditions [24,25]. Additionally, the composition of gut microbiota changes with age, in particular cases leading to a higher abundance of pro-inflammatory bacterial strains [26]. Defining eubiosis, a state of balanced microbiota composition, is therefore challenging [27]. Indisputably, eubiosis is the opposite of dysbiosis—the disturbed composition of microbiota that could cause both infectious and non-communicable diseases [25]. The gut microbiota plays a central role in physiological and pathological conditions and is the primary focus of most research [28]. Population-based cohort studies, such as the Metagenomes of the Human Intestinal Tract study (conducted in 2010 on 124 healthy European adults) or the Human Microbiome Project (conducted in 2010 on 242 healthy adults from the United States), have attempted to establish the typical composition of the human microbiome [29]. Approximately 90% of the gut microbiota consists of the two dominant phyla Firmicutes and Bacteroidetes [30], and the ratio of these phyla has been suggested as a marker for distinguishing between eubiosis and dysbiosis [31]. Furthermore, the gut microbiota appears to be an important regulator of healthy aging [32], while changes in its composition may be associated with CI serving as a potential biomarker [20]. Table 1 provides an overview of the molecular mechanisms by which gut microbiota can influence cognitive aging. The bidirectional interrelationship between the gastrointestinal tract and the CNS has long been studied, leading to the coining of the term ‘gut-brain axis’ that has further evolved into the ‘microbiota-gut-brain axis’, emphasizing the importance of gut microbiota in the inter-organ cooperation [33]. This axis involves complex interactions driven by neural connections through the vagus nerve, endocrine signals, and immune system mediators [34]. Age itself could affect this communication, potentially influencing microbiota composition [35]. However, a study by Bian et al. on a large cohort of over 1000 healthy Chinese individuals demonstrated that the microbiota composition of the healthy elderly group was similar to that of young people [36]. Hence, changes in microbiota composition may not be directly related to chronological age (measured in years), but rather to biological age [35], which can be assessed through frailty measurements [37]. Frailty is a complex syndrome that manifests itself through one’s functional status, influenced by many signs, symptoms or other health-related events [37]. A systematic review by Wen et al. showed significant differences in the gut microbiota composition between frail and non-frail elderly [38]. Moreover, frail elderly had reduced alpha diversity and significantly decreased abundance of certain microbiota genera, including Roseburia, Faecalibacterium, and Prevotella [38]. Roseburia spp. are commensals producing short-chain fatty acids (SCFAs), especially butyrate, which have anti-inflammatory properties. Moreover, Roseburia spp. are thought to be markers of health and are thought to be potential probiotics [39]. Figure 1 illustrates a flowchart showing how gut microbiota composition, whether healthy or pathological dysbiosis, may affect the aging process.

## 4. Immune System Modulation

Whether the fetus is colonized by maternal microbiota in utero or immediately after birth remains a subject of debate. Nonetheless, research emphasizes that children born through natural labor and breastfed tend to develop diverse and healthy microbiota [40]. There is a growing body of evidence that the maternal gut microbiota drives fetal immune system programming [41]. Finally, in the first three years of life, the composition of the gut microbiota evolves to a configuration similar to that of an adult, which is accompanied by maturation of the host immune system [42].

Under normal physiological conditions, the host immune system effectively sustains immune tolerance, balancing the relationship between the host and commensal bacteria [43]. This balanced interaction is regulated by the action of intestinal epithelial cells, mucus, secreted immunoglobulin (Ig) A, antimicrobial peptides and immune cells, collectively referred to as the ‘mucosal firewall’ [44]. Mucus secreted by goblet cells forms two layers, the inner of which is dense, and its role is to reduce the direct interaction of intestinal microorganisms with the gastrointestinal epithelium [45]. If bacteria breach the intestinal barrier, dendritic cells (DCs) located in the lamina propria capture them and transport them to the mesenteric lymph nodes, where DCs serve as antigen-presenting cells for T and B cells [46]. Naïve T cells, influenced by antigen presentation, differentiate into regulatory T cells that produce anti-inflammatory cytokines. B cells initiate IgA production into the intestinal lumen [34]. IgA constitutes a crucial component of the host immune response, preventing mucosal infections [47]. On the contrary, dysbiosis could trigger an immune response and a further release of pro-inflammatory cytokines [34].

A study by Qi et al. comparing two cohorts of healthy adults differentiated by age showed that compared to the younger cohort, adults over 70 years of age had significantly elevated levels of zonulin, a marker of increased intestinal permeability, and of high-mobility group box protein, which triggers inflammation [48]. Additionally, an animal-based study by Thevaranjan et al. demonstrated that under germ-free conditions, mice did not exhibit an age-related increase in pro-inflammatory cytokine concentrations [49]. This suggests that age-related dysbiosis may be the triggering factor for the inflammatory response and its subsequent consequences [49]. Figure 1 illustrates a flowchart showing how gut microbiota composition, whether healthy or pathological dysbiosis, may affect the aging process.

## 5. Direct Neuroinflammation or Inflammaging?

The blood-brain barrier (BBB) plays a vital role in maintaining the CNS isolation from the peripheral circulation. It primarily regulates the entry of substances into the CNS and facilitates the removal of metabolites from it [50]. The BBB consists of endothelial cells connected by tight junctions, together with pericytes and astrocyte end-feet. These components work together to maintain the integrity and functionality of the barrier [51]. Disruption of the BBB is a component of healthy aging and does not necessarily involve neurodegeneration. However, when accompanied by factors such as neuroinflammation, BBB dysfunction may contribute to cognitive decline [52]. Additionally, recent studies suggest that the composition of the gut microbiota might influence BBB permeability, as dysbiosis can lead to decreased expression of tight junction proteins [53]. Moreover, dysbiotic bacteria are detected by pattern recognition receptors on the host immune cells, which then activate the innate immune response. Toxins released by these pathogens can damage epithelial cells, leading to increased intestinal permeability and allowing pathogens to enter the circulation [34]. Pro-inflammatory cytokines released into the bloodstream eventually reach the BBB [54] where they can compromise its permeability [55]. For instance, tumor necrosis factor (TNF)-α has been proven to reduce BBB integrity in an in vitro model [56]. Increased BBB permeability enables peripheral immune cells to infiltrate the CNS. Consequently, the peripheral immune response may be a contributing factor in the development of Alzheimer’s disease (AD) [57]. A study in a mouse model of episodic systemic inflammation showed that age-associated CI may be related to neuroinflammation and oxidative stress [58]. Chronic systemic inflammation is closely associated with inflammaging [59], which is a condition that can lead to changes in the gut microbiota over time [60]. Inflammaging is associated with elevated levels of serum pro-inflammatory cytokines, including TNF-α, interleukin (IL)-6 or C-reactive protein (CRP), while levels of anti-inflammatory cytokines, such as IL-10, are reduced [61]. The elevation of pro-inflammatory cytokines has been associated with an increased risk of vascular diseases, which in turn may contribute to cognitive decline in the elderly [62]. A study by Cattaneo et al. focused on analyzing the association between brain amyloidosis in CI elderly and specific gut microbiota taxa with established pro-inflammatory or anti-inflammatory properties. Additionally, the authors aimed to identify peripheral circulating cytokines associated with AD pathogenesis. The presence of amyloid was associated with a higher abundance of the pro-inflammatory *Escherichia*/*Shigella* and a lower abundance of the anti-inflammatory *Eubacterium rectale* in stool samples compared to healthy controls and CI patients without amyloid deposition [63]. Therefore, there appears to be an association between gut microbiota composition, inflammatory parameters and cognitive status in the elderly [21].

## 6. Short-Chain Fatty Acids and Neuroprotection

The gut microbiota is capable of transforming dietary components into a variety of metabolites, including SCFAs, trimethylamines, amino acid derivatives, or vitamins. These metabolites play a crucial role in maintaining homeostasis, but some of them may also contribute to pathological processes [54]. SCFAs, such as acetate, butyrate, and propionate, are primary metabolites synthesized in the colon through bacterial fermentation of dietary fiber and undigested remains. These SCFAs are crucial for maintaining intestinal barrier integrity, stimulating mucus production, and providing protection against inflammation [64]. According to a study by Arpaia et al. in a mouse model, butyrate and propionate were found to stimulate the generation of anti-inflammatory Treg cells in the periphery [65]. They are also considered key modulators of the gut-brain axis [64]. SCFAs act primarily through two pathways: one involves activation of G protein-coupled receptors (GPCRs), and the other is associated with inhibition of histone deacetylases (HDACs). GPR43 and GPR41, also known as free fatty acid receptor 2 (FFAR2) and FFAR3, respectively, along with GPR109A, also known as HCAR2, are expressed in a variety of cells, including those in the CNS [66]. GPR43/FFAR2 is responsible for the anti-inflammatory effect of SCFAs in the CNS, while GPR109A plays a potential role in maintaining BBB integrity [67]. On the other hand, a decrease in SCFA levels and the abundance of bacteria producing them could lead to various pathologies. According to a study conducted by Zhang et al. in a mouse model of AD, *Ruminococcus* and *Butyricicoccus* genera were significantly decreased in 8–12-month-old AD mice compared to age-matched controls. Moreover, the concentrations of SCFAs were significantly lower in both feces and brains of AD mice [68]. This preclinical research is consistent with a cross-sectional study by Salazar et al., where the authors observed that aging was associated with a progressive and statistically significant decrease in fecal concentrations of SCFAs [69]. Ultimately, SCFAs may serve as neuroprotective agents. According to Liu et al., *Clostridium butyricum*, a butyrate-producing bacterium, significantly reduced cognitive dysfunction and histopathologic changes in mice subjected to permanent unilateral right common carotid artery occlusion, a model used to study vascular CI [70].

## 7. Oxidative Stress

Oxidative damage is one of the hallmarks of brain aging, as noted by Mattson and Arumugam [8]. The mitochondrial theory of aging, particularly important in the context of ROS-induced damage, emphasizes reduced oxidative capacity. It has been proposed that mutations in mitochondrial DNA can alter the expression of oxidative phosphorylation complexes, initiating a vicious circle of oxidative capacity and increased ROS production, further contributing to cellular aging [71]. Oxidative stress and the cellular damage it causes are involved in age-dependent cognitive dysfunction. Therefore, oxidative stress is not only a marker of aging, but also a significant contributor to age-related pathologies by inducing cellular and molecular damage [72]. Recent studies have pointed to the role of the gut microbiota as a potential player in oxidative stress levels [73]. For instance, components of the gut microbiota, such as Lactobacillus, Bifidobacterium, Streptococcus or Bacillus, have the ability to produce nitric oxide (NO). Elevated NO levels may lead to the formation of reactive oxygen and nitrogen species. These species can cause neuroinflammation and axonal degeneration, thereby providing a link between gut microbes and neurodegenerative diseases [74]. Additionally, research by Mossad et al. revealed that a metabolite produced by the microbiota, known as N6-carboxymethyllysine, accumulates in the microglia of aging mice. This metabolite triggers an increase in ROS, potentially leading to increased oxidative stress damage [75].

## 8. Oral Microbiota and Neurodegeneration

The term oral microbiota refers to the variety of microorganisms colonizing the oral cavity [76], ranking second in microbial abundance within the human body [77]. A study by Aas et al. demonstrated that the predominant genera of typical oral microbiota include *Gemella*, *Granulicatella*, *Streptococcus*, and *Veillonella* [78]. Oral microbiota usually form a biofilm, whose main role is to maintain oral homeostasis and prevent disease development [77]. On the other hand, dysbiosis of the oral microbiota triggers periodontal diseases, such as periodontitis and gingivitis, which are known to be associated with other systemic diseases [79]. Recently, oral microbiota dysbiosis has also been associated with neurodegenerative diseases, including AD [80]. The pathways linking oral microbiota composition changes with neurodegenerative conditions are summarized in Table 2. A systematic review by Said-Sadier et al. found that periodontitis may be associated with CI but the exact pathomechanism remains unclear [81]. Among several pathogenetic theories of AD, inflammation is one of the main mechanisms driving the progression of the disease [82]. As the prevalence of periodontal disease increases with age and is highest in individuals aged 65 years or over [83], periodontitis-induced low-grade inflammation may be a link between oral dysbiosis and age-related CI [84]. Also, *Porphyromonas gingivalis*—a Gram-negative, obligate anaerobic bacterium that is a major contributor to periodontal disease [85], has been found to be associated with CI [86]. A study by Poole et al. on brain specimens obtained at autopsy from 10 AD patients and 10 non-AD controls found lipopolysaccharide from *Porphyromonas gingivalis* in AD individuals but not in controls [87]. The most significant virulence factors of *Porphyromonas gingivalis* are cysteine proteases called gingipains R and K, which specifically cleave after the amino acids arginine and lysine, respectively. Furthermore, there are two types of gingipain R (RgpA and RgpB) and only one type of gingipain K (Kgp) [85]. Dominy et al., showed that RgpB and Kgp levels were significantly elevated in AD brains compared to non-AD control brains and correlated with tau protein levels. Moreover, the authors demonstrated neurotoxic effects of gingipains on tau proteins in vitro as well as in vivo in an animal model, suggesting small-molecule inhibitors of gingipains as a potential treatment method. Finally, the gingipain inhibition reduced amyloid-β1–42 production and decreased neuroinflammation as measured by the levels of TNF-α levels [88].

## 9. Limitations

This review describes the potential role of gut microbiota and oral microbiota in age-related cognitive decline, emphasizing primarily general mechanisms and established interactions between host and microbes. Thus, the review covers a broad and complex topic. Due to the extensive scope of this subject, this paper does not exhaustively cover all its aspects. For instance, the variability of microbiota across different races, continents, or regions is not discussed. Additionally, elderly patients take numerous medications, which might also influence the composition of the microbiota, thereby potentially influencing cognitive health. However, these aspects represent extensive areas of research that require focused studies due to their complexity.

## 10. Conclusions and Future Research Perspectives

In summary, emerging evidence supports the role of the microbiota in maintaining homeostasis, as well as in the development of disorders, particularly those associated with aging, such as neurodegenerative diseases. Although most research to date has focused on the gut microbiota, commensals residing in the oral cavity may also influence age-related cognitive disorders [89]. Generally, dysbiosis induces low-grade chronic inflammation that spreads through the bloodstream to all body tissues, triggering systemic changes. Within the CNS, dysbiosis is associated with the disruption of the BBB and its increased permeability, subsequently leading to neuroinflammation. Gut microbiota may induce oxidative stress, which is considered to be one of the hallmarks of brain aging. Nevertheless, due to limited amount of longitudinal studies, any causal inferences regarding microbiota and specific cellular mechanisms must be interpreted with caution.

Research describing gut microbiota composition changes associated with aging, is inconsistent and requires further investigation. In particular, future studies should focus on the role of commensal and pathogenic microorganisms associated with biological aging rather than a chronological measure of years. Finally, there is a strong need for randomized clinical trials investigating the therapeutic potential of microbiota restitution or supplementation with microbial metabolites, such as SCFAs, in slowing biological aging and/or treatment of age-related diseases.

## Figures and Tables

**Table 1:** Potential Molecular Mechanisms by which Gut Microbiota Influence Cognitive Aging.

Process	Description	The Impact on Cognitive Aging
Dysbiosis-driven intestinal barrier dysfunction	Gut microbiota dysbiosis impairs intestinal tight junctions, increasing the permeability of intestinal wall, allowing toxins and pathogen inflow into systemic circulation	Exposure to microbial antigens and toxins triggers systemic inflammation and subsequent neuroinflammation
Immune system modulation and inflammaging	Persistent low-grade inflammation with circulating cytokines triggers microglial activation	Neuroinflammation disrupts synaptic function and heightens vulnerability to cognitive decline
Blood-brain barrier disruption	Pro-inflammatory cytokines compromise blood-brain barrier integrity, permitting the infiltration of toxins and peripheral immune cells into the central nervous system	The increased blood-brain barrier permeability leads to neuroinflammation and neuronal damage, that exacerbate age-associated cognitive impairment
Decrease in short-chain fatty acids production	Decrease in gut microbiota genera responsible for short-chain fatty acid production has been linked to the process of biological aging (as measured by frailty)	Short-chain fatty acids are thought to have neuroprotective effects. Their decreased levels diminish these effects, increasing susceptibility to neurodegenerative processes
Oxidative stress	Dysbiosis contributes to excessive reactive oxygen species production and decreased antioxidative capacities	Accumulating oxidative damage impairs neuronal function, compounding age-related cognitive impairment

**Table 2:** Potential Role of Oral Microbiota in Age-Associated Neurodegenerative Disorders.

Oral Condition	Molecular Mechanisms	Association with Neurodegeneration
Oral dysbiosis	Shifts in oral microbiota composition, leading to biofilm disturbances and subsequent periodontal disorders	Increased risk of cognitive impairment and neurodegenerative disorders. Facilitates systemic inflammation that may be associated with Alzheimer’s disease pathophysiology
Chronic periodontitis	Chronic systemic inflammation associated with local oral inflammatory processes as well as possible hematogenous spread of oral pathogens	Correlation between periodontitis and cognitive impairment incidence. Also, periodontal diseases are thought to exacerbate Alzheimer’s disease
*Porphyromonas gingivalis* colonization	Crosses the blood–brain barrier, triggering neuroinflammation, and secretes gingipains contributing to tau protein alteration and amyloid-β production	Neuroinflammatory cascade aggravating cognitive decline by amyloid deposition and tau pathology
Co-existence with traditional cognitive impairment risk factors	Shared risk factors (e.g., lower educational level, smoking, poor hygiene) and synergistic effects of diabetes or smoking on vascular and inflammatory pathways	Susceptibility to vascular dementia and Alzheimer’s disease

## Data Availability

No new data were generated.
